# When the lung invades: a review of avian postcranial skeletal pneumaticity

**DOI:** 10.1098/rstb.2023.0427

**Published:** 2025-02-27

**Authors:** Andrew J. Moore, Emma R. Schachner

**Affiliations:** ^1^ Department of Anatomical Sciences, Stony Brook University, Health Sciences Center,101 Nicolls Road, Level 4, Stony Brook, NY 11794-8081, USA; ^2^ Department of Physiological Sciences, University of Florida, 1333 Center Drive, Gainesville, FL 32608, USA

**Keywords:** pneumaticity, bird, air sac, dinosaur, bone architecture, biomechanics

## Abstract

Birds are unique among extant tetrapods in exhibiting air-filled cavities that arise from the respiratory system and invade postcranial bones, a phenomenon called postcranial skeletal pneumaticity (PSP). These intraosseous cavities originate from diverticula of the ventilatory air sacs or directly from the gas-exchanging lung. Despite a long history of study, many of the basic characteristics of this system remain poorly understood. In this hybrid review, we synthesize insights from the anatomical, developmental, biomechanical and paleontological literature to review the functional and evolutionary significance of PSP. Leveraging new data, we confirm that the skeletons of pneumatic birds are not less heavy for their mass than those of apneumatic birds. Pneumatic skeletons may nonetheless be lightweight with respect to body volume, but this is a hypothesis that remains to be empirically tested. We also use micro-computed tomography scanning and deep learning-based segmentation to produce a pilot model of the pneumatized spaces in the neck of a Mallard (*Anas platyrhynchos*). This approach facilitates accurate modelling of bone architecture for quantitative comparative analysis within and between pneumatic taxa. Future work on PSP should focus on the cellular mechanisms and developmental processes that govern the onset and extent of pneumatization, which are essentially unknown.

This article is part of the theme issue ‘The biology of the avian respiratory system’.

## 1. Introduction

Among living tetrapods, birds are unique in exhibiting postcranial skeletal pneumaticity (PSP), which results from the invasion of bone and the resorption of bone tissue and marrow by diverticula of the gas-exchanging lung and its ventilatory air sacs [1–4]. However, PSP arose much earlier than the avian crown clade, among the ornithodiran (i.e. pterosaurs + non-avian dinosaurs) relatives of extant birds [5–7]. In addition, PSP has been proposed to occur in the vertebrae of some osteoglossomorph fishes as a result of extensions of the respiratory gas bladder [8,9], but a causal role for the bladder in producing cavernous spaces in these vertebrae remains to be demonstrated.

In this review, we integrate palaeobiological, developmental, biomechanical and anatomical data on skeletal pneumaticity within an evolutionary framework, with an emphasis on the impact of pneumaticity on the functional properties of bone. We also evaluate the merits of competing hypotheses for variation in extent of pneumaticity. For the purposes of clarity, we term any intraosseous diverticulum a *pneumatizing diverticulum*, owing to its presumed causal role in producing air-filled spaces within the bone. We apply this term to any pulmonary diverticulum that invades bone, regardless of whether it is a *parabronchial diverticulum* (*new term*; a diverticulum that emerges directly from the gas-exchanging lung) or *saccular diverticulum* (*new term*; a diverticulum that arises from a ventilatory air sac). These latter terms are not exhaustive, and pneumatizing pulmonary diverticula may potentially arise from other sites (e.g. a secondary bronchus).

In several respects, craniofacial air sinuses remain better-studied than those of the postcranial skeleton, and some paradigmatic ideas about the function and development of pneumatic spaces were developed with regard to paranasal air sinuses. For these reasons, we make occasional recourse to studies on cranial pneumaticity. We note, however, that treating cranial and postcranial pneumaticity as essentially equivalent phenomena, as is common in the pneumaticity literature (e.g. [10,11]), is an untested assumption that remains to be investigated by comparative developmental studies.

## 2. Anatomical and phylogenetic distribution of postcranial skeletal pneumaticity

### (a) Patterns of postcranial skeletal pneumaticity in extant birds

Extant birds show considerable variation in the relative extent of PSP between different species and across the skeleton of a single individual [1,2,4,11–29]. Large, clade-specific surveys of the regional extent of pneumaticity [23,25,27,28] indicate that within-clade variation in PSP is greatest in groups that make substantial use of aquatic environments (Anseriformes, Aequorlitornithes), which include several entirely or nearly apneumatic lineages. In contrast, essentially terrestrial clades (Cuculidae, Accipitrimorphae) have comparatively static and extensive patterns of pneumaticity, encompassing most of the postaxial vertebral column, humerus, sternum and pelvic girdle, among other elements [27,28]. Secondary diminishment or complete loss of PSP occurs numerous times within Neornithes (e.g. penguins, auks, diving ducks, darters), where it is in most cases conspicuously associated with an aquatic foraging lifestyle [14,15,22–25,29]. At the other extreme, various birds (e.g. New and Old World vultures, pelicans, screamers, bustards, storks and hornbills) exhibit a ‘hyperpneumatic’ phenotype in which most of the bones of the skeleton are aerated [15,24]. At least some of this substantial interspecific variation in PSP can be explained by differences in body size and locomotory ecology (see ‘§7’).

**Figure 1.**
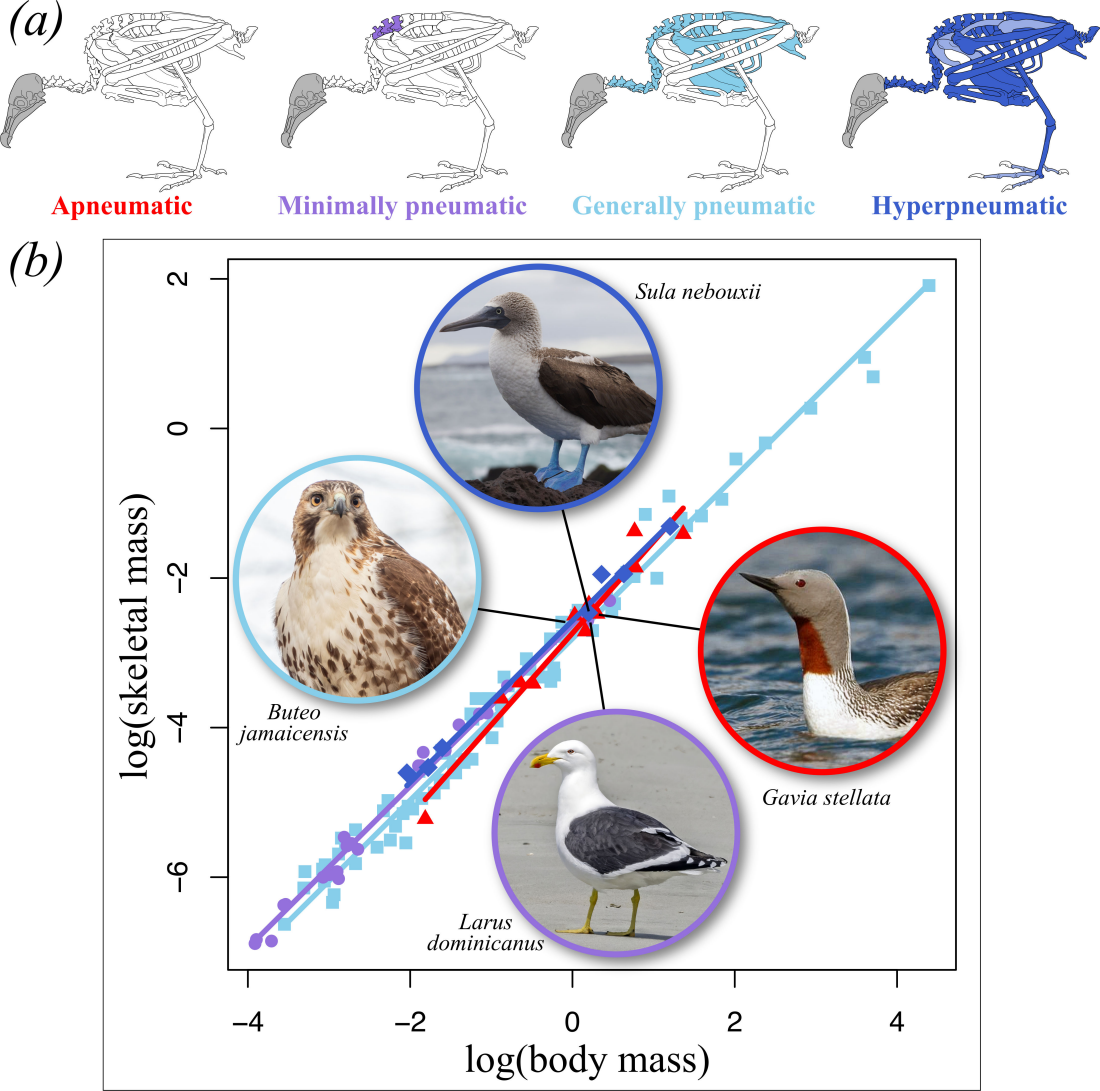
Scaling of skeletal mass with body mass in extant birds across varying extents of PSP. (*a*) Skeletal illustrations depicting four gross categories along a continuum of variation in extent of PSP, inspired by fig. 3 of [24]. (*b*) The scaling relationship between log-transformed body mass and skeletal mass for birds of varying degrees of pneumaticity. Pneumatic and apneumatic birds show statistically identical scaling relationships and they broadly overlap in bivariate trait space: the apneumatic *Gavia stellata*, minimally pneumatic *Larus dominicanus*, generally pneumatic *Buteo jamaicensis*, and hyperpneumatic *Sula nebouxii* fall close together on the plot, with a skeletal mass ranging from 6.2% (*G. stellata*) to 7.4% (*B. jamaicensis*) of body mass. Image attributions: *Gavia stellata*–Ómar Runólfsson, CC BY 2.0 DEED; *Larus dominicanus*–Charles J. Sharp, CC BY-SA 4.0 DEED; *Buteo jamaicensis*–Rhododendrites, CC BY-SA 4.0 DEED; *Sula nebouxii*–Ndecam, CC BY 2.0 DEED.

Apneumatic and hyperpneumatic birds are end members of a pneumaticity continuum (figure 1*a*). Between these two extremes, O’Connor [23,24] identified a ‘floor’ on the extent of PSP (herein termed the ‘minimum pattern’) that serves as a clade-wide baseline in extent of PSP. The minimum pattern includes pneumatization of vertebrae surrounding the cervicothoracic junction, and typifies various shorebirds, some ducks and at least some neoavians ([23,24], this study). Decreases from the minimum pattern result in an apneumatic skeleton (e.g. kiwi, penguins, loons and grebes), whereas increased pneumaticity typically encompasses the remaining precaudal and postaxial vertebrae and their ribs, as well as the sternum, pelvic girdle and humerus. O’Connor [24] termed this the ‘extended pattern’, but because it is the predominant condition across those avian species that have been sampled to date (with additional clade-specific variation in elements like the axis, caudal vertebrae, shoulder girdle and femur; [23,24,27,28], this study), we refer to it as the ‘general pattern’. Finally, increased PSP beyond the general pattern characterizes hyperpneumatic birds and includes some otherwise rarely pneumatized bones such as distal limb elements and the pygostyle.

The existence of a minimum baseline and a loosely hierarchical progression in overall extent of pneumaticity constrains the combinations of pneumatic anatomical units observed in birds. For example, all examined taxa with pneumatic scapulae also exhibit pneumatic cervicothoracic vertebrae, and taxa without pneumatic cervicothoracic vertebrae lack pneumaticity in all other anatomical units ([23,24,27,28], this study). Similarly constrained patterns of evolutionary variation in PSP also occur in non-avian theropod dinosaurs [6], perhaps suggesting the existence of canalized pathways by which evolutionary change in pneumaticity proceeds.

Intraspecific variation in extent of pneumaticity can be substantial, both in terms of which elements are pneumatized (e.g. [27]) and asymmetry in the location of pneumatic features (e.g. [30]). For example, the humeri of adult chickens (*Gallus gallus*) were apneumatic in 11/48 (23%) of the specimens sampled by Hogg [21], and unilaterally pneumatized in 14/37 birds (38%), among similar variations (see also [17]). The factors that produce such variation remain poorly understood, and could include age, sex and pathological or physiological processes (e.g. sexual activity) [17,18,26], as well as the generally opportunistic—and thus stochastic—nature of pneumatizing epithelia [10,31]. Although intraspecific variation in PSP has been quantified for a few species (e.g. [18,21,26,32,33]), these studies were not designed to test hypotheses about the factors that predict that variation, and some lack controls on sex. Qualitative data on variation in pneumaticity in *G. gallus* [17,21] could suggest that males are more pneumatic than females and that female sexual activity status has no significant impact on pneumaticity extent, but the data do not lend themselves to statistical testing.

Although older treatments of avian pneumaticity often mention the specific bony targets invaded by pneumatizing diverticula of particular air sacs (e.g. [2,16,19,20,22,34–36]), work by O’Connor and Claessens [37] and O’Connor [4] has been central to establishing the paradigm that only certain components of the avian pulmonary system invade the skeleton, and do so in invariant and prescribed ways. Specifically, these authors argued that only three components of the pulmonary system participate in pneumatizing the axial skeleton, with consistent targets: (i) cervical air sac diverticula invade the cervical and cranialmost thoracic vertebrae and their ribs; (ii) abdominal air sac diverticula pneumatize post-mid-thoracic, synsacral and caudal vertebrae and the pelvic girdle; and (iii) the gas-exchanging lung itself gives off parabronchial diverticula that invest adjacent thoracic vertebrae and ribs with air. In addition, the interclavicular air sac has been found to pneumatize the sternum and its ribs, the shoulder girdle and the forelimb (e.g. [2,35]), with distal forelimb elements in some large-bodied soaring birds being pneumatized by subcutaneous diverticula of the interclavicular sac [4,24]. The cranial thoracic sacs occasionally aerate sternal ribs (e.g. [36]), whereas the caudal thoracic air sacs are described as lacking pneumatizing diverticula (e.g. [2,35]).

The universality of these patterns is challenged by the recent computed tomography (CT) scan-based reconstruction of the lung in *Struthio camelus* [38]. This study identified a previously unappreciated role for parabronchial diverticula in directly pneumatizing the femur and the entirety of the precaudal vertebral column (see also [39]), raising the possibility that putatively fixed patterns of pneumatization are not as conserved as they have been described to be. Additional testing via CT scanning, latex injection and gross dissection of the ‘rules’ proposed by O’Connor and Claessens [37] and O’Connor [4] is an area of ongoing research. To that end, high-resolution micro-computed tomography (µCT) scanning of freshly frozen deceased specimens whose respiratory systems have been artificially inflated provides the best data for non-destructively visualizing the respiratory system and tracing its diverticula with high fidelity [40]. Gross dissection and latex injection casts remain important complementary approaches for validating inferences made from CT scans. However, because these methods are inherently destructive, they are most appropriate for addressing highly specific research aims—for example, establishing the utility of CT scanning for assessing the presence or absence of a particular diverticulum [41], or evaluating the morphology of the horizontal septum of the avian lung [42].

### (b) Recognizing postcranial skeletal pneumaticity in extant and fossil ornithodirans

**Figure 2.**
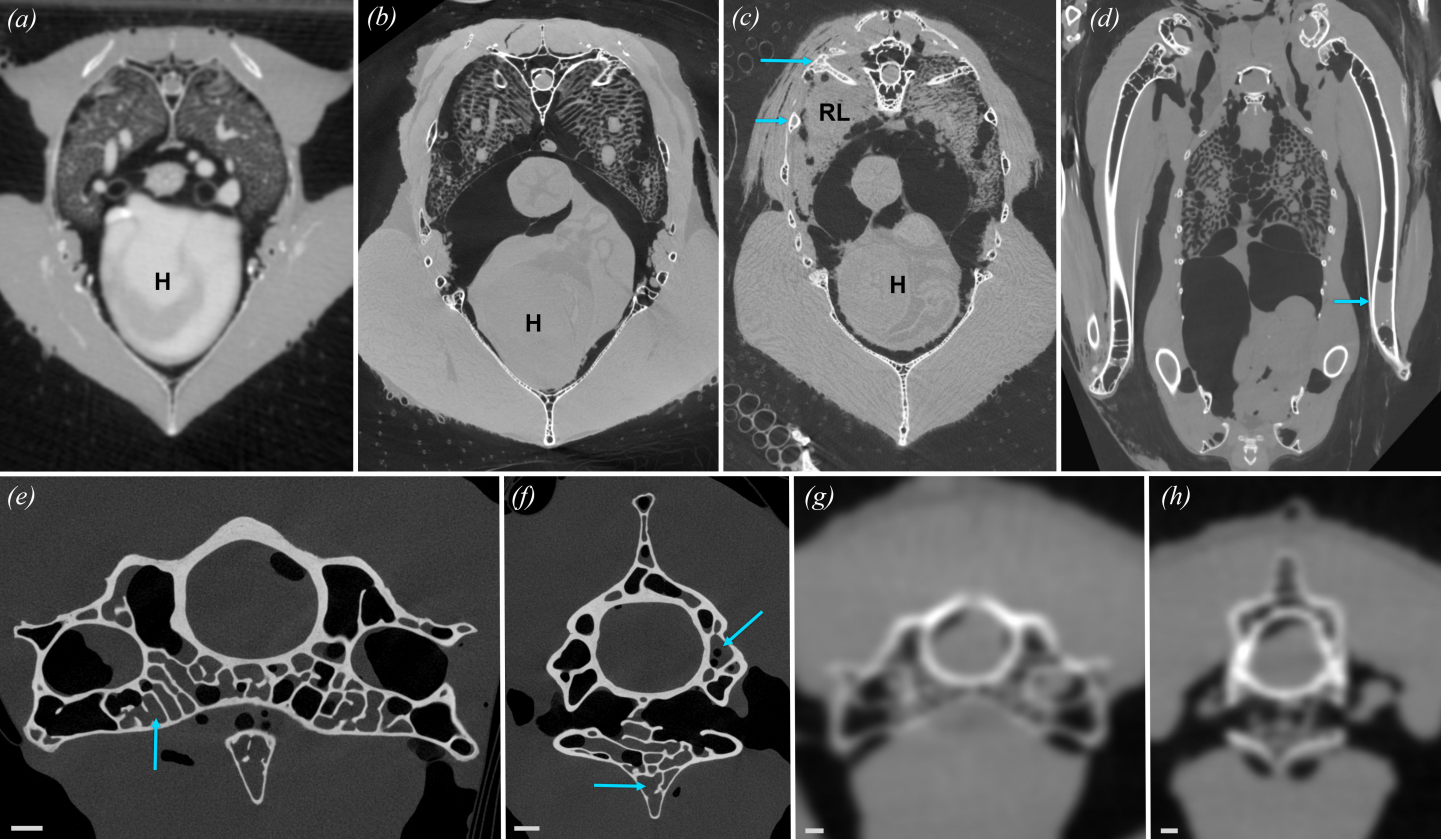
µCT images of birds demonstrating the impact of decay on the ability to evaluate pneumaticity status in a specimen. (*a*) Axial section of the thorax in a live sedated female Swainson’s Hawk (*Buteo swainsoni*). Axial µCT section of the thorax of (*b*) a deceased and artificially inflated red-tailed hawk (*Buteo jamaicensis*; sex unknown) with no pulmonary disease or decay and (*c*) a *B. jamaicensis* with severe asymmetric decay that dramatically and unilaterally impacts the pneumatic spaces. (*d*) Coronal µCT section of the humeri, coelomic cavity, pelvis and femora of a deceased and artificially inflated eastern screech owl (*Megascops asio*; sex unknown) showing asymmetric accumulation of fluids in the humerus owing to slight decay (blue arrow). Axial µCT slices of cervical vertebra 14 (C14) of a deceased male wild mallard (*Anas platyrhynchos*) at (*e*) cranial and (*f*) slightly more caudal positions after multiple freeze–thaw cycles and extended periods of time at room temperature, demonstrating asymmetric accumulation of fluids in typically pneumatized spaces. (*g, h*) Axial µCT slices of similar locations in C14 of a live sedated male *A. platyrhynchos* showing the lack of fluid buildup observed in (*e, f*); although resolution is necessarily lower in live scans, scrolling through the µCT stack makes it clear that this vertebra is wholly pneumatic, or nearly so. All blue arrows indicate fluid and decay in pneumatic spaces that are typically filled with air in living specimens. Scale bars, 1 cm. H, heart; RL, rotten lung.

Identifying PSP in a living or freshly frozen deceased bird is straightforward if the specimen has been CT scanned at sufficient resolution, because radiolucent intraosseous air spaces are unambiguous (figure 2*a*,*b*). However, most previous work on pneumaticity has been conducted on defleshed skeletal or fossil specimens, necessitating the identification of robust osteological correlates of PSP. In a seminal study, O’Connor [4] established a hierarchy of soft tissue specificity for several commonly cited correlates of pneumaticity, demonstrating that a relatively large cortical opening leading to large internal chambers within the bone is the only unambiguous gross correlate of PSP (see also [5,43]). All other gross osteological features provide less clear evidence because they can also be associated with other non-pulmonary soft tissues (e.g. vasculature, muscle, fat). The presence of unambiguous correlates of PSP in sauropodomorph (e.g. [5,44–49]) and theropod dinosaurs (e.g. [5,6,50,51]) and pterosaurs (e.g. [5,52–56]) provides direct anatomical evidence for a heterogeneous pulmonary structure in these taxa, and suggests that a pulmonary soft tissue system capable of producing PSP—minimally, a heterogeneous lung with a flexible, caudoventral sac-like component—is ancestral for Ornithodira [4–7,38,42,57].

Pneumatizing epithelia may also leave a distinctive osteohistological signature. For cranial and postcranial bones in avian and sauropod specimens, Lambertz *et al*. [58] found that pneumatized bone is characterized by fine, densely packed parallel fibres that are distinguishable from the thicker Sharpey’s fibres left by muscle attachments, and termed this tissue type ‘pneumosteum’. Interestingly, they failed to find pneumosteum in the craniofacial sinuses of mammals, suggesting clade-specific signatures of pneumatizing epithelia. Although additional work is needed to establish the conditions under which pneumosteum can be confidently identified, osteohistology shows promise for recognizing PSP in fossil taxa that otherwise bear only ambiguous gross correlates of PSP (e.g. [59]).

## 3. Quantifying pneumaticity

### (a) Discretized anatomy

Most quantitative studies on the evolution of PSP have discretized focal taxa into a series of anatomical compartments [6,23,24,27,28,60] or pneumatic characters [25] and measured extent of PSP as a fraction of total anatomical units exhibiting pneumaticity (e.g. figure 2 of Benson *et al*. [6]). This Pneumaticity Index (PI; [23]) has the benefits of relying on relatively simple data and allowing direct, quantitative comparisons between individuals. Previous studies using the PI have provided important insights into the influence of body mass and ecology on the gross extent of PSP in extant birds and their extinct ornithodiran relatives [6,23–25,27,28] (see ‘§7’). However, the PI is an inherently coarse metric. It is concerned only with the presence or absence of pneumaticity within a given anatomical unit, and thus treats a bone that is 44% air by volume as equivalent to one that is more than 80% air (i.e. the range of variation observed in the presynsacral vertebrae of storks; [11]). Similarly, the PI makes no distinction between skeletal regions of dramatically different sizes (e.g. the humerus versus caudal vertebrae) and thus starkly divergent potentials to impact whole-body mass or other functionally relevant factors through their pneumatization. Moreover, summarizing whole-organism extent of pneumaticity as a fractional metric may homologize anatomically—and perhaps functionally—distinct patterns of pneumaticity [25], potentially obscuring evolutionary variation in PSP and complicating interpretations of correlational analyses and ancestral state reconstructions.

### (b) Air space proportion

Most studies to quantify extent of pneumaticity for a single bone have used the air space proportion (ASP; [61]), which is the ratio of the space occupied by air to the total (i.e. air + bone) volume [11,29,61–67] (e.g. figure 1 of Moore [11]). Previous studies using the ASP have made the reasonable assumption that the volume occupied by the pneumatic epithelia is negligible, given that these epithelia are exceedingly thin membranes [3,68]. Potentially more problematic for studies of defleshed or fossil specimens is the common assumption that bone marrow within the medullary space is wholly absent; if islands of marrow persist in non-negligible volumes [17,18,32,69], ASP values will be artifactually inflated.

In a recent study of humeral pneumaticity, Burton *et al*. [29] measured the persistence of marrow by exclusively sampling whole (i.e. intact) frozen specimens and quantifying three volumes: bone volume, air volume and intraosseous soft tissue (i.e. marrow) volume. These volumes were then used to calculate two versions of the ASP, which together provide a more nuanced view of the real extent of air spaces within pneumatic bones (see also [67]).

While accounting for the persistence of marrow is important for inferences about the impact of PSP on bone density and biomechanical behaviour [70,71], the Burton *et al*. [29] study lacks controls on the time between death and freezing, and hence on the level of decay. Our work indicates that decay-related, water-density substances can accumulate rapidly within the respiratory system (i.e. within 8 h postmortem; [40]) and typically manifest asymmetrically (figure 2*c*,*d*). This conclusion is supported by substantial anecdotal evidence demonstrating that regions of the skeleton that are wholly pneumatic in live birds become increasingly infiltrated with water-density tissues in deceased birds that were scanned multiple times post-mortem (figure 2*e*–h; E.R.S. personal observation, 2023). Moreover, even in instances in which decay has been negligible, freeze–thaw cycles can cause the accumulation of water-density substances within pneumatic spaces that can be shown in live scans to lack them. Without exacting control over specimen provenance and care, the appearance of water-density soft tissues within intraosseous air spaces cannot be assumed to accurately represent conditions during life. We therefore strongly recommend that quantitative studies of PSP be restricted to the use of specimens frozen immediately postmortem (< 1−2 h).

### (c) Measures of bone architecture

The PI and ASP metrics provide intuitive measures of the gross extent of PSP across the skeleton or within a bone and they are useful for comparing broadscale patterns in PSP. However, these metrics fail to characterize the structural and biomechanical impact of pneumaticity on cortical and trabecular bone tissues. These features are instead best quantified using established bone architectural measurements with hypothesized relationships to bone loading and mechanical properties (reviewed in [71,72]). Particularly important among these are trabecular bone volume fraction and degree of trabecular anisotropy because these predict bone strength and stiffness, but other metrics (e.g. trabecular connectivity; average cortical shell thickness) are also useful [71–73]. Comparative studies using these metrics are a promising but underexploited [26,33] area for future research on the functional significance of avian PSP.

## 4. Development and maintenance of pneumatic spaces

The physical and biochemical mechanisms underlying variation in the presence, developmental timing and extent of skeletal pneumatization remain almost entirely unknown [24]. Several descriptive histological studies have characterized the ontogenetic development of cranial (e.g. [74–78]) and postcranial [3] pneumatic spaces. The genetic basis of pneumatic phenotypes has been interrogated through transcriptomic comparison of pneumatizing and non-pneumatizing epithelia in the rodent middle ear [79,80] and through study of the heritability of primate craniofacial air sinus morphology [81,82]. However, beyond these limited forays, the intrinsic and epigenetic systems governing skeletal pneumaticity are largely unexplored.

Pneumatizing epithelia are commonly posited to impact bone pneumatization by influencing the activity of osteoclasts and osteoblasts at the site of the epithelium–bone interaction [10,24,31,75,77,80,83], but the mechanisms by which this occurs remain very poorly understood. The adjacency of a pulmonary diverticulum to the cortical surface of a bone is insufficient on its own to bring about PSP. For example, in various species of apneumatic birds (e.g. auks; [24]), pneumatizing diverticula may be appressed to the postcranial skeleton without inducing pneumatization, indicating that PSP is not purely a function of the relative development of diverticula [24]. This suggests that gene expression or the activity of other biochemical regulators are involved in pneumatization, but the identities and mechanisms of these molecular actors remain unknown. Bremer [3] suggested a role for maternally derived oestrogens, inspired by the hypothesis that these hormones induce the development of an intraosseous ‘mesenchymal pathway’ for the pneumatizing epithelium to follow within the chicken humerus. Nitric oxide (NO), a potent signalling molecule, may play a role in mammalian craniofacial pneumaticity: the paranasal sinuses are a major endogenous source of respiratory NO [84] and this gas is implicated in sinus wall remodelling through its effects on osteoclast activity ([85] and references therein). Studies of craniofacial sinuses suggest that genetics may be an important determinant of the pneumatic phenotype [81,82], but characterization of the specific genes involved is in its infancy [79,80,86] and has yet to be extended to postcranial pneumatization.

Detailed data on the relative and absolute timing of postcranial skeletal pneumatization are sparse. Published accounts exist only for turkeys (*Meleagris gallopavo*; [87,88]), domestic chickens (*G. gallus*; [32]) and pigeons (*Columba livia*; [89]) and are supplemented by unpublished dissertations on budgerigars (*Melopsittacus undulatus*; [90]) and quail (*Coturnix coturnis*; [91]). At hatching, the avian postcranial skeleton is entirely apneumatic, its medullary spaces filled with haematopoietic marrow [32,87,89]. In *M. gallopavo, G. gallus* and *C. livia*, postcranial pneumatization does not commence until several weeks post-hatching (approx. day 28−35), when bone ossification is complete [89]. The presynsacral vertebrae are among the first bones pneumatized, but specific pneumatization patterns differ across these three species. In *G. gallus*, the mid-region of the neck and the humerus are the first parts of the skeleton to be pneumatized, and post-axial cervical pneumatization reaches completion before the onset of thoracic or synsacral aeration, both of which commence around day 77, typically with the caudal thoracic vertebrae and cranial synsacral vertebrae [32]. In contrast, the cranial thoracic vertebrae of *C. livia* are aerated before any other regions of the skeleton; by three months (the next known time point), pneumaticity invests much of the postcranial skeleton [89]. Like *C. livia*, the thoracic vertebrae of *M. gallopavo* (as well as the sternum) are pneumatized before other precaudal vertebrae [87]. Despite the limited nature of the data, the divergent trajectories in these species demonstrate that ontogenetic pneumatization patterns are not strictly conserved across birds, and that axial pneumatization may occur in cranial-to-caudal or caudal-to-cranial directions (*contra* [92]). Importantly, the comparatively well-resolved sequence for *G. gallus* cannot be taken as representative of birds as a whole or cited as evidence for developmental recapitulation of evolutionary patterns observed in non-avian ornithodirans (*contra* [6,7,47,48,61,92,93]). Establishing the relative conservatism of postcranial pneumatization patterns will require concerted sampling of the ontogenetic trajectories of a taxonomically diverse sample of birds.

Almost nothing is known about the intrinsic and extrinsic factors that influence the timing and sequence of postcranial pneumatization. Experimental undernourishment brings about earlier onset of humeral pneumatization in chickens [94]. The rate of humeral pneumatization in white leghorn chickens appears to be greater in female than male individuals, suggesting possible sex-specific differences [21]. Most studies indicate that patency of the pneumatic foramen is required to develop and maintain intraosseous pneumatic spaces. Disruption of this connection halts the process of pneumatization (e.g. [95] and references therein, [96–98]; but see [86,99]) and can ultimately obliterate the original pneumatic chamber [95,97,98]. Although intraosseous pneumaticity and medullary bone can sometimes jointly occupy the medullary cavity in egg-laying female birds, the predominant pattern is an inverse relationship, whereby the ephemeral deposition of medullary bone tissue does not occur in pneumatized bones [100]. Bremer [3] provides the only detailed histological study of the process by which normal postcranial pneumatization unfolds, focusing on the humerus of *G. gallus* from 12−20 days post-hatching. By the time the growing axillary diverticulum—a derivative of the interclavicular sac—enters the humerus through a mesenchyme-filled channel occupied by nutrient veins, progressive resorption of trabecula has already begun. Degeneration of marrow occurs not only for those masses that become surrounded by pneumatizing epithelium, but also those enveloped solely by mesenchyme. This observation might accord with the hypothesis that the disappearance of marrow is related to the regression of the capillaries that feed it, rather than to some particular absorptive capacity of the diverticulum itself [16]. Ultimately, the branches of the invading diverticulum fuse anastomotically to form a single large intraosseous air space, limiting any remaining marrow to the ends of the shaft. Interestingly, recent data from the humeri of developing wild turkeys suggest that, after an initial air space expansion, relative air volumes may decrease ontogenetically, with a ‘bounce back’ in relative marrow volume [88].

The observation that pneumatizing diverticula often share a common foramen with neurovascular structures has led to the hypothesis that pneumatization generally requires the pre-existence of perforating nutrient blood vessels [3–5,101]. The topological correspondence between nutrient foramina in the vertebral centra of non-pneumatic archosaurs and unambiguously pneumatic foramina in birds and other ornithodirans provides additional support for this hypothesis [4,6]. However, the extent of this dependency, and the developmental mechanisms by which diverticula might take advantage of a pre-existing vascular ‘highway,’ are unknown.

## 5. Pneumaticity and bone biomechanics

Higher-order influences on the gross extent of pneumaticity are somewhat better characterized than fine-scale developmental processes. In particular, the onset and maintenance of PSP are often described as dynamic and opportunistic processes, mediated in part by the loading environment of the bone so as to keep biomechanical stresses within functional limits (reviewed by [96]; see also [2,69,102,103]). This model predicts that pneumatizing diverticula operate as ‘opportunistic pneumatizing machines’ [96, p.64], actively invading a bone and preferentially causing bone resorption in those areas that experience the least stress or that lack functionally important structures like teeth (e.g. [2,5,75–77,96,102]). The extent of pneumaticity in any given element is hypothesized to reflect a dynamic tension between the pneumatizing drive of the epithelium and the tendency for deposition of new bone in response to loading demands ([96]; but see [31,81,104]). Under this view, minimum-mass structural solutions to particular biomechanical challenges emerge as an inherent product of the system [96].

Direct empirical tests of this hypothesis are rare and we are aware of only a single study to demonstrate that experimentally diminished loading results in significant bone resorption through expansion of a pneumatic sinus (in this case, the maxillary sinus of recently edentulous dogs; [105]). However, indirect evidence for this view can be found in the observation that serial variation in vertebral pneumaticity is consistent with shape- and locus-specific biomechanical expectations [11,26]; that the most highly pneumatic portions of sauropod cervical vertebrae are under the least stress under hypothetical loading conditions [106]; that the distal limb elements of dynamic soarers (which navigate highly energetic settings) are apneumatic whereas those of static soarers (which experience slow-moving thermals) are typically pneumatic [24]; and that the osprey (*Pandion haliaetus*) is the only accipitrimorph to exhibit apneumatic femora and conspicuously engages in feet-first, high-angle diving into water [28] (see [11,96] for additional examples). We discuss the possible biomechanical benefits and consequences of PSP below (see ‘§7’).

## 6. Impact of pneumaticity on bone architecture and material properties

Few studies have directly assessed the effect of PSP on the structural and material properties of bone, from theoretical or empirical perspectives. These studies generally fall into two categories: those investigating vertebrae, where standard bone architectural metrics are compared across vertebral positions and species, and those focusing on long bones, with an emphasis on the relationship of pneumaticity to relative cortical thickness.

### (a) Vertebrae

Vertebrae are the most commonly pneumatized bones and they are thus a critical locus of inquiry for understanding the impact of pneumaticity on bone architecture. To our knowledge, only two studies have explicitly characterized bone architectural properties in avian vertebrae. Fajardo *et al*. [33] showed that the pneumatic third thoracic centrum of the wood duck (*Aix sponsa*) has significantly lower whole bone volume fractions (i.e. higher ASP values) than the apneumatic centrum of the ruddy duck (*Oxyura jamaicensis*). This difference results primarily from the significantly thinner cortices of the pneumatic vertebrae. In contrast, mean relative trabecular bone volumes are indistinguishable between pneumatic and apneumatic centra, although trabeculae are somewhat thinner and more numerous in the pneumatic *A. sponsa* [33]. Gutzwiller *et al*. [26] compared the vertebral centra of apneumatic and pneumatic representatives of Pelecaniformes and Charadriiformes. They found that cortical bone was significantly thinner in the vertebrae of the highly pneumatic brown pelican (*Pelecanus occidentalis*) than in the apneumatic Anhinga (*Anhinga anhinga*) and double-crested cormorant (*Phalacrocorax auritus*), similar to the ducks studied by Fajardo *et al*. [33]. In contrast, apneumatic and pneumatic charadriiforms did not significantly differ from one another in either cortical thickness or relative trabecular bone volume. Considered together, these data suggest that PSP often results in thinner cortical bone and underscore the potential significance of element-, clade- and ecology-specific factors in determining bone architecture and extent of pneumaticity.

### (b) Long bones

Several studies [70,71,107,108] have developed theoretical arguments to explain the existence of tubular long bones in amniotes, under the assumption that tubular bones are designed to save mass within the context of a particular function (i.e. optimization for yield strength, fracture strength, impact strength or stiffness) and have modelled expected differences in diaphyseal cross-section for marrow-filled versus pneumatic bones. Calculations for minimum-mass marrow-filled tubular bones must take into account the mass of the marrow, the volume of which grows as the bone walls become increasingly thin. Optimizing such bones for yield or fatigue strength predicts a diaphyseal radius, *R*, three times greater than wall thickness, *t* (i.e. an *R*/*t* value of 3.0), which amounts to a mass saving of about 13% over a solid bone ([70,71]; see also [109]).

Pneumatic long bones, on the other hand, can be modelled as filled only with gas of negligible mass (but see [29]). If optimized solely for yield strength in response to bending, pneumatic long bones would be expected to have infinite radii and vanishingly thin walls. Such a design is clearly unrealistic, however, not least because it ignores the risk of failure by buckling. For a given bending moment, the cross-sectional area needed to avoid failure by buckling is proportional to (*R*/*t*)^1/3^ [70,107]. Thus, increasingly thin-walled bones are constrained to increase in cross-sectional area (and therefore mass) to avoid failure due to buckling. For pneumatic bones, Alexander [108] argued that the theoretically optimal situation is one in which the masses for failure under both bending and buckling are the same, and estimated that this condition is met for cross-sections with approximately *R*/*t* = 14. This specific value may be inaccurate owing to simplified assumptions regarding the elastic properties of bone. Nevertheless, the optimal *R*/*t* value for air-filled bones is expected to be higher than for marrow-filled bones [108].

To assess the correspondence between theoretical expectations and empirical reality, Currey and Alexander [70] gathered *R*/*t* values for a sample of 228 bones from 56 species of amniotes, mostly comprising terrestrial mammals, bats and birds; these data have since been supplemented for birds by several other studies [110–112]. Avian long bones are generally thinner-walled than those of mammals (see also [34]); this suggests that birds—whether pneumatic or apneumatic—are under greater selection than are mammals for reduced mass [70], assuming that bone density is essentially constant across the sample (but see [113]). For apneumatic bird bones, measures of central tendency of the *R*/*t* value fall close to the predicted minimum mass optima for static strength (3.0) and stiffness (3.9) of marrow-filled bones [70,111]. The range of *R*/*t* values observed in pneumatic bones (3.1−7.1) overlaps without exceeding the higher end of the range observed in apneumatic bones (1.5−7.1) [70,110–112]. Thus, while pneumatic avian long bones are, on average, thinner-walled than apneumatic bones [29,111], pneumaticity does not fundamentally alter or expand the cross-sectional shape space exploited by pneumatic avian lineages. Notably, the most extreme *R*/*t* value observed in birds (7.1) falls far below the predicted value of *R*/*t* = 14 for minimum-mass pneumatic bones optimized for static strength [114].

These results for birds contrast starkly with the highly pneumatic pterosaur *Pteranodon*, the only animal for which *R*/*t* values approached or exceeded theoretical expectations for a minimum-mass design for a pneumatic bone (e.g. *R*/*t* = 20 for humerus and ulna), suggesting divergent selective pressures. Whereas the forelimb long bones of *Pteranodon* indeed appear to have been pushed to the brink to save mass, the comparatively thick walls of pneumatic bird bones are not maximally constructed for mass reduction, and instead appear to be engineered to provide resistance to occasional direct blows [70]. Importantly, such high *R*/*t* values for *Pteranodon* are only feasible in pneumatic bones: a marrow-filled bone of the same cross-sectional shape would in fact be *heavier* than a marrow-filled bone with a more modest *R*/*t* value [108,109].

Cubo and Casinos [111] extended the experimental approach of Currey and Alexander [70] to include explicit mechanical testing of pneumatic and apneumatic avian long bones. Apneumatic bones were found to be significantly stronger and stiffer than pneumatic bones (see also [12]). Taken at face value, these results suggest that long bone pneumatization entails a real sacrifice to bone integrity [111]. But if pneumaticity can make bones weaker, why pneumatize them at all? Answering this question requires consideration of the potential selective advantages of PSP.

## 7. Adaptive benefits of postcranial skeletal pneumaticity

The biomechanical model of skeletal pneumatization outlined above is potentially consistent with two alternative views on the adaptive benefits of PSP: that it improves energetics through holistic or regional reductions in mass, or that it improves bone biomechanics by optimizing bone architecture in mechanically advantageous ways. It has also occasionally been suggested that PSP is involved in thermoregulation by increasing the respiratory surface area available for evaporative cooling (e.g. [5,115]). While it is possible that the highly compliant air sacs and their diverticula indeed assist in thermoregulation (briefly reviewed in [42]), it seems unlikely that the intraosseous air spaces are capable of contributing appreciably to lung ventilation or evaporative heat loss, given that the stiffness of a bone prevents its active participation in intraosseous air circulation [15,61], and we do not consider this hypothesis further.

### (a) Improved energetics through reduction in skeletal mass and density

By far the most commonly advanced adaptive hypothesis for PSP is that it produces energetic benefits by reducing skeletal mass and density [1,5,6,24,25,29,34,43,46,70,116]. Skeletal pneumatization replaces marrow and bone tissue—especially dense cortical bone [26,33,70,111]—with air, and is therefore expected to lower the energetic cost of movement for pneumatic birds and other ornithodirans [4,24]. Such savings are potentially advantageous at any size [6,24,43]. However, they are thought to be particularly important for flying taxa ([5,22,34,43,56,70,102,117] but see [69]), for which locomotion is energetically costly, and for large-bodied taxa [24,46,56,106,118], given that the mass saved by the removal of marrow should approximately scale with the cube of linear bone size, making removal of marrow and bone tissue increasingly advantageous at larger sizes [15].

Despite the pervasiveness of the hypothesis, very few studies have attempted to quantify mass savings resulting from PSP. Campana [69] expressed scepticism that pneumaticity provides meaningful mass reduction, and estimated PSP to reduce body mass in *Gallus* by a mere 0.7−0.8%, an amount dwarfed by normal physiological mass fluctuations (see also [90]). Estimates for non-avian dinosaurs have been more generous. Wedel [61] proposed that PSP lightened the body mass of the sauropod *Diplodocus* by *ca* 1455 kg, or 8−10% vis-à-vis a hypothetically apneumatic skeleton, and Witmer and Ridgely [119] found that cranial pneumaticity lightened the heads of two theropods by 7−8% (where head mass was approximated by assigning specific tissue masses to regions of a hypothetical volumetric model). Although such modest savings may appear inconsequential [109], Currey and Alexander [70] argued strongly that mass reduction on the order of *ca* 10% could amount to significant, selectively advantageous savings to the energetic cost of locomotion.

The repeated evolutionary loss of PSP in diving birds can also be understood as relating to the optimization of locomotory energetics, in this case by modulating body density through the removal of incompressible air spaces. Accompanied by the relative thickening of the cortical wall [29,33,120,121], the apneumatic bones of diving birds lower buoyancy resistance and thus improve the energetics of underwater foraging [15,20,23–25,29,122–125]. Thus, body size and locomotory mode are commonly invoked as primary factors influencing variation in the extent of PSP [20,22,24,25].

Despite the intuitive allure of the idea that larger-bodied (non-diving) birds and other ornithodirans should be more pneumatic, this hypothesis has generally garnered weak support from empirical studies that have sought to test it [6,11,23–25,27–29,60]. Recent studies on the wholly terrestrial avian clades Cuculidae [27] and Accipitrimorphae [28] have yielded particularly reserved support, as both clades exhibit high ‘baseline’ levels of PSP that leave fewer variably pneumatized anatomical units available to be explained by variation in body size. Interestingly, Benson *et al*. [6] found evidence of lower body size thresholds for the evolution of high PI values in non-avian theropod groups more closely related to birds. This suggests that while extent of pneumaticity may have initially evolved as a response to the gravitational constraints imposed by large masses, it became increasingly adaptive in near-avian maniraptoran lineages with increasingly high inferred metabolic rates [6,126,127], underscoring the potential for clade-specific thresholds for a given extent of PSP.

Notably, all of the foregoing studies quantified the extent of PSP using some form of PI, and thus the relatively weak predictive power of the positive correlations that they recovered may be due, at least in part, to the inherent coarseness of the metric. For example, the 24 species of the duck genus *Anas* sampled by O’Connor [23] were nearly identical in their PI values, despite a 10-fold difference in body mass between the smallest and largest species. In this case, it is probable that real differences in the extent of pneumaticity exist between individuals that would be captured if a metric with greater nuance (i.e. ASP or bone architectural measures) were applied to the quantification of PSP, as has been demonstrated for stork species spanning a comparable range in body mass [11]. For this reason, the PI is perhaps best-suited for identifying major shifts away from typical clade expression patterns, especially those related to particular ecological and locomotory specializations like diving and soaring [28].

Studies that use more holistic and precise measures of PSP can provide novel views on the relationship between body size and PSP [11,29]. For example, focusing on serial and interspecific variation in ASP in the presynsacral vertebrae of storks, Moore [11] found that size is an imperfect predictor of extent of pneumaticity, applicable only to certain loci. He instead argued that bone biomechanics—as reflected indirectly by the shape and serial position of a vertebra—have primacy in determining extent of pneumaticity, with greater ASP values occurring in vertebrae that are predicted to have more consistent and predictable loading regimes that, in turn, make them more amenable to a lightweight build.

Several studies have considered the potential influence of low-density respiratory volumes on whole-body centre of mass—an important parameter for studies of locomotion and biomechanics—with mixed results. In sauropod dinosaurs, high ASP values in the cervical and dorsal vertebrae produce a relatively caudal position of whole-body centre of mass compared with models with lower ASP values for these segments [128], indicating that mass savings resulting from PSP might have important impacts on the static and locomotory biomechanics of the unique sauropod *bauplan*. In contrast, volumetric studies on birds have mostly failed to identify substantial changes in centre of mass when comparing different sets of hypothetical segment densities [129–131]. These divergent results suggest that the impact of PSP on segment mass and whole-body centre of mass may be sensitive to variation in body shape, with only minor influences on avian centre of mass, but more work explicitly modelling pulmonary air spaces and specific soft tissue densities in birds is needed.

Considered collectively, previous work supports the hypothesis that PSP is an adaptation for holistic body mass reduction. However, the general weakness of the relationship, and the substantial intra- and interspecific variation in PSP across birds and other pneumatic ornithodirans, suggest that element-, clade- and ecology-specific factors play more proximate roles in determining extent of pneumaticity.

#### (i) Are pneumatic skeletons actually less massive than apneumatic skeletons?

The hypothesis that pneumaticity lightens the skeleton is potentially challenged by empirical work demonstrating that bird skeletons are not lighter than those of mammals of the same mass ([113,132]; see also [69,102,133]). Prange *et al*. [132] found that the scaling relationship between skeletal mass and body mass in a phylogenetically diverse sample of birds is statistically indistinguishable from that in mammals, with skeletal mass scaling with slightly positive allometry in both groups. Dumont [113] found similar results in a comparison between passerine birds and rodents. One shortcoming of these studies, however, is that they treat birds as uniformly pneumatic: neither study differentiates apneumatic or nearly apneumatic birds (e.g. penguins, cormorants, grebes) from birds that pneumatize much of their skeletons (e.g. pelicans, accipitrimorphs, storks), leaving open the possibility that the reported avian scaling relationship in each study in fact comprises two separate but closely subparallel regimes, with pneumatic birds potentially having a lower intercept and thus lighter skeleton.

To assess this possibility, we gathered data on regional pneumatization for 118 of the 209 species included in the Prange *et al*. [132] dataset, focusing exclusively on non-passeriforms and drawing on previous studies [23–25,27,28] as well as new data for 79 species. For six of these, data were taken from a congener or close relative of a species in the Prange *et al*. [132] dataset. Where Prange *et al*. [132] collected skeletal and body masses for multiple individuals per species, we summarized these values as a species average. Each sampled species was assigned a pneumaticity status for each of three sets of categories that treat whole-skeleton extent of pneumaticity in different ways. In the ‘Strict’ set, birds were considered pneumatic if they showed any evidence of PSP in any element, and were otherwise categorized as apneumatic. In the ‘Loose’ set, birds with the minimum pattern were instead grouped with wholly apneumatic birds. Finally, in the ‘Spectrum’ set, birds were assigned to one of four categories that characterize a continuous gradient of PSP: (i) apneumatic, (ii) the minimum pattern, (iii) the general pattern, and (iv) hyperpneumatic (figure 1*a*). We then used phylogenetically informed regression and phylogenetic analysis of covariance [134] on a time-calibrated phylogeny (see [41] for details) to assess (i) whether skeletal mass scales with isometry or allometry in different PSP categories, and (ii) whether groups of birds with different degrees of PSP differ in the scaling of their skeletal mass. All code and input files necessary to reproduce these analyses are provided in the electronic supplementary material.

Our results bear out the conclusions of Prange *et al*. [132] and Dumont [113]: despite the superficially delicate and ‘lightweight’ appearance of bird bones, in the aggregate, avian skeletons weigh as much as those of (non-chiropteran) mammals of similar body mass, irrespective of the extent to which they are pneumatized. We found no evidence for differential scaling of skeletal mass in pneumatic birds (table 1), which broadly overlap apneumatic birds in bivariate trait space (figure 1*b*). In agreement with Prange *et al*. [132], our phylogenetically informed regressions strongly support positive allometric scaling of the skeleton: regardless of pneumaticity status, more massive birds have relatively heavier skeletons (slope = 1.08; 95% CI = 1.05, 1.11).

**Table 1.** Results of least squares phylogenetic analysis of covariance tests assessing whether gross differences in extent of postcranial skeletal pneumaticity affect the scaling relationship between skeletal mass and body mass. Entries in the test column indicate which scaling coefficients were varied, against the null hypothesis of a single, global scaling relationship applicable to all birds. See text for details.

	test	degrees of freedom	sum of squares	mean sum of squares	*f*-value	*p-value*
global scaling		2	6.7303	0.0581		
pneumatic versus apneumatic (strict)	intercept	3	6.7392	0.0586	0.0014	0.9700
pneumatic versus apneumatic (strict)	slope and intercept	4	6.4966	0.057	2.1300	0.1235

						
global scaling		2	6.7393	0.0581		
pneumatic versus apneumatic (loose)	intercept	3	6.6437	0.0578	1.6544	0.2009

						
global scaling		2	6.7393	0.0581		
pneumatic versus apneumatic (spectrum)	slope (apneumatic different) and intercept (all different)	6	6.2705	0.056	2.0936	0.0863

Several hypotheses can be advanced to explain the incongruence between these results and the perception that bird skeletons are lightweight. Prange *et al*. [132] proposed that, instead of holistic reductions in mass, pneumaticity redistributes bone mass across the skeleton and thus provides a mechanism for producing functionally significant regional heterogeneity in bone density and segment mass. For example, progressive pneumatization of the axial skeleton may have improved bipedal agility and energy efficiency in non-avian theropods by reducing rotational inertia and torque [135]. The same is likely true for some of the largest and longest-necked sauropods, which have mostly apneumatic limb girdles and long bones but which can possess cervical vertebrae with ASP values as great as or greater than those of living birds [11,61,64,136].

An alternative explanation for the delicate-but-heavy paradox is that bird bones are, on the whole, denser than those of mammals [113]. To test this possibility, Dumont [113] compared cranial, humeral and femoral densities of passerine birds with those of bats and rodents of similar masses. Considering these elements *en masse*, birds have the densest bones of the three groups, suggesting that their skeletons are lightweight in the sense that they have larger strength-to-weight and stiffness-to-weight ratios than small mammals [113]. However, this pattern is driven by the cranium, which is significantly denser (and thus stiffer) than the skulls of small mammals. In contrast, passerine femora were significantly less dense than those of mammals, and humeral densities were indistinguishable across the three groups, although birds showed greater variance in humeral density. Problematically, the pneumaticity status of the long bones was not considered, and with few exceptions [29,110,137], the extent of PSP in passeriforms is largely unknown. Thus, the relationship—if any—between extent of PSP and bone tissue density in passeriforms and other birds remains to be tested.

The superficially surprising observation that pneumatic bird skeletons are as massive as those of apneumatic taxa may not, in fact, be paradoxical. Removal of bone tissue by pneumatizing diverticula without a concomitant reduction in the magnitude of forces acting on a pneumatic bone will result in applied forces being distributed over a smaller cross-sectional area (i.e. greater stress), and thus an increased risk of bone failure [11]. This situation would seem to demand that pneumatization be accompanied by structural reorganization of pneumatic body segments to prevent dangerous sacrifices to bone integrity. The finding that pneumatic bird skeletons are as heavy for their body masses as those of apneumatic birds and mammals (figure 1*b*) suggests that pneumatic savings in bone mass occur in lockstep with commensurate reductions in soft tissue mass, keeping the relative skeletal mass of pneumatic birds in line with the scaling relationship for the apneumatic taxa. Much of these soft tissue savings are expected to result from the loss of marrow mass. It is also possible that some of these savings accrue from reductions in the muscle mass necessary to move and manipulate the pneumatized skeleton, although the limited available data on the scaling of muscle mass in birds [138] might argue against this possibility, at least for some body segments. Importantly, lower soft tissue mass would have the effect of diminishing the static compressional forces and torques acting on pneumatic bones, while reductions in muscle mass (if they occur) would also reduce the muscular forces applied to pneumatic body segments.

If this is indeed the case, then the skeletons of pneumatic birds are not light compared with body *mass*, but they may instead be light with respect to body *volume*. Comparing hypothetical birds of equal whole-body volume that differ only in their expression of PSP, the pneumatic bird is expected to be less massive than the apneumatic bird, owing, at a minimum, to the removal of bone tissue and bone marrow by pneumatizing epithelia (this assumes that bone density is unchanged in the pneumatic bird—an assumption that requires additional testing). Thus, the pneumatic bird is volumetrically larger for its mass than is the apneumatic bird. Such decoupling of the volume : mass relationships that characterize apneumatic tetrapods is hypothesized to release birds and other pneumatic ornithodirans from structural constraints on body size evolution, allowing these animals to be volumetrically larger for their mass [24]. Direct tests of this hypothesis have yet to be conducted, but could be accomplished using large comparative datasets of high-fidelity volume and mass data for birds and other sauropsids [131,139].

For pneumatic ornithodirans experiencing particularly strong selection pressure for reduced mass, it seems probable that other structural or behavioural modifications have evolved to permit increased pneumatization. For example, a relatively diminished range of motion should produce a less variable and more predictable loading regime that is, in turn, more amenable to the minimalist construction produced by extensive pneumatization [11,26]. In this light, the relatively stiff necks of the largest sauropods may represent a structural accommodation to the evolution of extremely long necks, whereby some flexibility is sacrificed for gains in absolute and relative length [11,136]. It is possible that a similar tension between extent of PSP and range of motion exists in extant birds, where this hypothesis is more readily testable.

### (b) Optimization of bone architecture

It is sometimes argued that pneumaticity produces skeletons that are equally good or better than apneumatic skeletons at resisting the forces applied to them (e.g. [1,15,27,28,34,102,106,116,119]). If pneumaticity allows *inflation* of a bone of a given mass by increasing its total (i.e. air + bone) volume, it would be expected to improve resistance to bending or torsion by placing bone material farther from the centre of the applied force (i.e. by increasing the second moment of area or polar moment of inertia; [15,27,28,102,111,116,119]). The same logic has been used to argue that air sacs serve to expand the size of the thorax without increasing its mass, thereby improving the mechanics of the trunk as a whole [2]. The hypothesized inflationary effect of PSP might also improve musculoskeletal biomechanics by increasing the attachment area [28] or lever arm of muscles that attach to external surfaces of pneumatic bones.

The inflationary potential of pneumatizing epithelia is reasonably well-established, at least in certain contexts (e.g. [79,80,85,96]; but see [103]). Various amniotes with cranial pneumaticity exhibit inflated bones (e.g. the auditory bulla in desert mammals; the pterygoid bulla in gharials; the hyoid of howler monkeys; [46,96,140–142]). At least in some cases, these may become inflated as a result of increased intrabulla air pressure, which promotes intramural resorption and extramural apposition of bone [85]. However, under normal conditions, these bullae are presumably under relatively minimal load and their inflation cannot be interpreted as producing biomechanically superior bone architecture of the kind suggested for load-bearing bones of the postcranial skeleton. Instead, these inflated pneumatic structures serve other purposes, like improving low-frequency audition [140,141] or enhancing vocalization by acting as a resonating chamber [46]. At present, it is not known whether pneumatization inflates load-bearing postcranial bones.

There are two potential problems with the hypothesis that pneumaticity serves to inflate a bone so as to improve its biomechanics. The first is theoretical. If a bone of a given mass is inflated, it will enjoy greater resistance to bending or torsion, but will nevertheless be made more susceptible to failure by buckling [71]. To avoid this risk, there are two structural solutions: increase bone stiffness (i.e. by laying down denser cortical bone), or increase bone cross-sectional area [71]. Both of these solutions require increases in bone mass. Thus, assuming an increased risk of failure by buckling is intolerable, pneumatic inflation of a bone will necessarily require an overall *increase* in skeletal mass. It is not obvious why such bones would be selectively advantageous over uninflated bones, especially given that this outcome (i.e. more massive bones) is antagonistic to the better-substantiated notion that pneumaticity offers energetic savings by reducing mass. That said, it is possible that the risks of failure by buckling could be mitigated by behavioural modification (e.g. favouring display over combat in intraspecies competition) or structural reorganization of pneumatic body segments, which might then make bone inflation viably advantageous.

The hypothesis that pneumaticity improves bone biomechanics is also hobbled by the difficulty of testing it. Comparing the architectural and mechanical properties of apneumatic and pneumatic bones is insufficient to demonstrate that biomechanically advantageous inflation has predominated over the possible benefits of mass reduction in determining bone structure [110]. Comparative histological and morphometric analysis of long bone cross-sections can illuminate structural features that are relatively more or less resistant to a particular kind of loading [110,121,143]. However, determining that any particular pneumatic bone has been either biomechanically improved or disadvantaged compared with how it would perform were it not pneumatized is challenging because of a dearth of information on the structural and loading differences that distinguish the pneumatic and hypothetically apneumatic conditions. Finite element analysis might offer a partial solution, as hypothetical apneumatic morphologies could be generated and tested against the observed, pneumatic condition. However, such comparisons are necessarily laden with assumptions about unobserved and unobservable bone architecture and loading conditions. This is particularly important in light of the hypothesis that pneumatic bones should experience different habitual loading compared with apneumatic bones because of reductions in their mass and possibly in the mass of the soft tissues necessary to manipulate pneumatic body segments. It should also be noted that different forms of loading [110,121,144], and different performance criteria [70] will influence optimal bone architecture.

The finding that pneumatic long bones are less strong in bending and less stiff than apneumatic bones lends superficial support to the view that skeletal pneumaticity imposes a risk, rather than a boon, to bone biomechanics [111,144], entailing a sacrifice to structural integrity to reap the energetic benefits of having a lighter skeleton. However, the extent to which this difference in mechanical performance actually matters for pneumatic birds remains unknown and can only be meaningfully assessed against experimental data on the modes and extremes of loading that they actually encounter. At present, such information is lacking for virtually all birds (but see [145–147], providing a rich area for future investigation.

## 8. Case study: high-resolution quantification of serial variation in axial pneumaticity

Large-scale comparative analysis of bone architecture and functional performance in pneumatic versus apneumatic bones will require the digital segmentation of large volumes of CT scan data, a historically time-consuming procedure demanding significant user input. Artificial intelligence (AI)-assisted CT scan segmentation offers a means of ‘high throughout’ processing that is fast, does not sacrifice accuracy and is reproducible by other researchers (e.g. [148–151]). We explored the potential of AI for rapid segmentation of whole specimens using deep learning-based tools in the software Dragonfly (v.2022.2; see https://www.theobjects.com/dragonfly), applied to the intact neck of a deceased adult male mallard (*A. platyrhynchos*; ERS2023−037 – Clovis). The entire cervical vertebral series was μCT scanned in seven overlapping segments at a resolution of 20 µm using a Waygate GE v|tomex|m machine at the Research Services Center of the Herbert Wertheim College of Engineering at the University of Florida (RRID:SCR_025135). The intensity scale was calibrated across scans to range from 0 (air) to 40 000 (bone). To produce training data, we manually segmented 26 slices from across the cervical series, capturing separate regions of interest for bone, soft tissue and air. These training data were further increased through 11 rounds of augmentation (default settings, plus Gaussian noise = 0.05). A U-Net semantic segmentation model was trained with default parameters, with 20% of the training data set aside for validation. A Dice coefficient of 0.988 indicated very high similarity of the U-Net segmentation to the training data. Following U-Net segmentation of each segment of the neck, we generated internal vertebral air volumes, following Moore [11]. Because of pervasive postmortem accumulation of soft tissue-density substances in spaces known to be wholly pneumatic in live mallards (figure 2), apneumatic intraosseous regions of the cervical skeleton were recognized by their lack of connection to unambiguously pneumatic chambers. These volumes were negligible except in cervical 1 (which is apneumatic) and cervical 6, which appears to lack neural arch pneumaticity. Pneumaticity was measured using the ASP_total_ metric [29].

**Figure 3.**
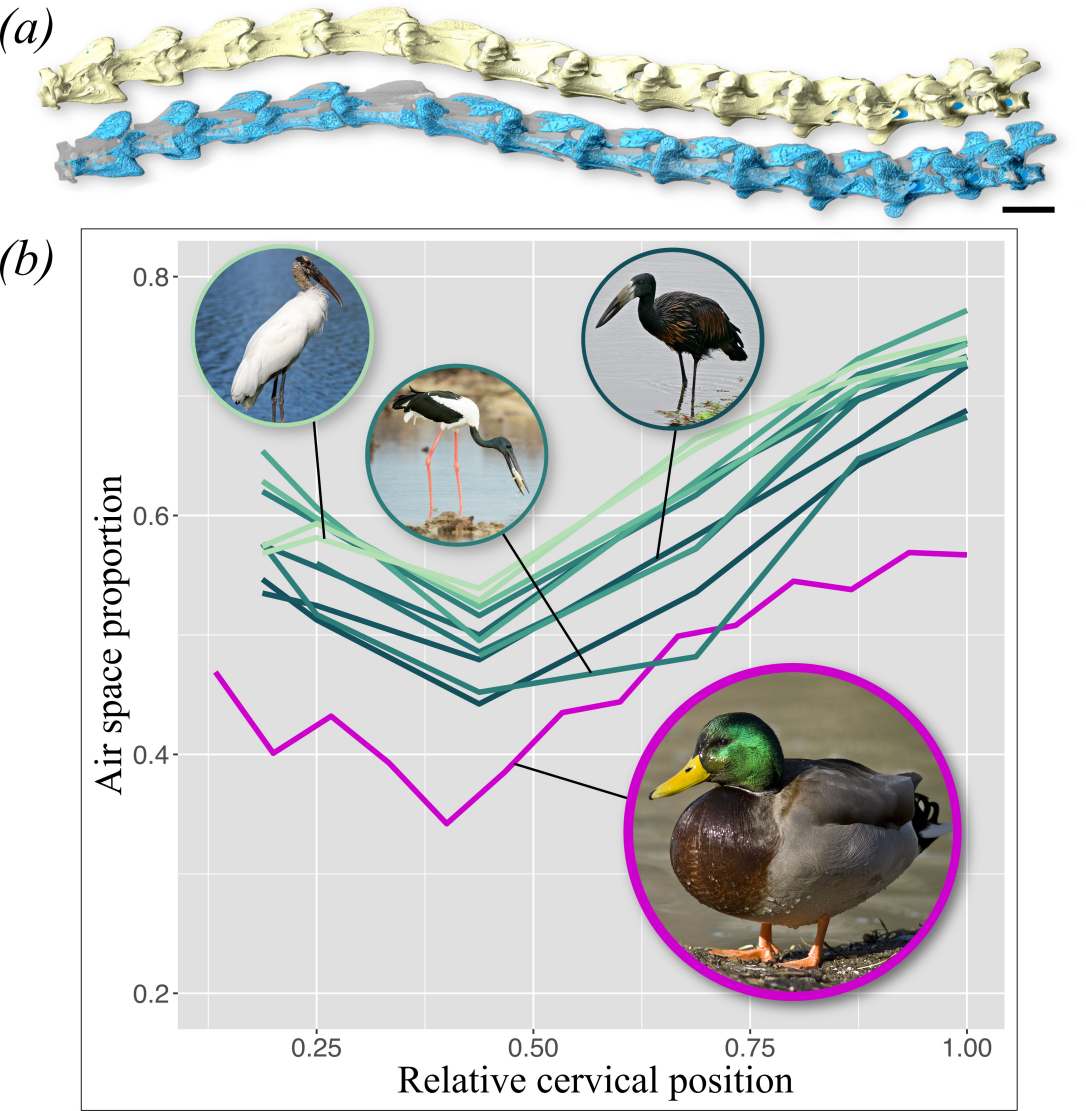
Serial variation in skeletal pneumaticity in the neck of Clovis (a specimen of *Anas platyrhynchos*) and various species of storks. (*a*) Left lateral view of the complete cervical vertebral series of Clovis, segmented using a U-Net model trained in the software Dragonfly (v.2022.2; see https://www.theobjects.com/dragonfly). In the lower row, bone has been made transparent to visualize serial variation in the volume of internal air space (blue). (*b*) Serial variation in ASP in Clovis and 11 species of storks sampled for cervical vertebrae 3, 4, 7, 11, 14 and 16 (from Moore [11]). Note that cervical 11 of the black-necked stork (*Ephippiorhynchus asiaticus*) is less pneumatic than comparable vertebrae in Clovis, despite being a much larger bird (body mass of approx. 4100 versus 749 g), underscoring that body size is an imperfect predictor of the extent of PSP. Image attributions: *Anas platyrhynchos* (reversed)—Alain Carpentier, CC BY 3.0 DEED; *Ephippiorhynchus asiaticus*—JJ Harrison; CC BY-SA 4.0 DEED; *Anastomus lamelligerus*—Bernard Dupont; CC BY-SA 2.0 DEED; *Mycteria americana*—Googie man; CC BY-SA 3.0 DEED.

U-Net segmentation correctly classes fine trabecular struts as bone and is thus more accurate than standard thresholding-based segmentation, which relies on grayscale values alone and often fails to capture thin trabeculae. Patterns of serial variation in ASP in *A. platyrhynchos* are similar to those in storks, but magnitudes are distinct (figure 3*b*; [11]). Cervical 11 of *Ephippiorhynchus asiaticus* is less pneumatic than comparable vertebrae in *A. platyrhynchos*, despite being a much larger bird (body mass of *ca* 4100 g versus 749 g), underscoring the imperfect correlation between body size and PSP [11].

## 9. Future directions

PSP is a hallmark innovation of birds and their extinct ornithodiran relatives, but despite more than a century of study, many of the fundamental characteristics of this system have yet to be elucidated. Continued work in several key areas will substantially improve our understanding of PSP.

Although gross regional patterns of PSP are now well-established for numerous neornithines, there is a near-total lack of data on the structural impact of pneumatization on avian bones. Characterizing how variation in the architecture of pneumatic bones manifests both intra- and interspecifically, and how these patterns compare with those observed in apneumatic birds and other sauropsids, will be central to understanding the functional significance of PSP. These efforts will require a standardized and efficient work flow for accurate recognition of pneumatic spaces and quantification of bone architecture. We propose that studies of PSP should be limited to live individuals and/or deceased, whole specimens for which the time between death and freezing is 1−2 h, as these practices should help mitigate spurious identification of intraosseous soft tissues. We have also demonstrated the potential of artificial intelligence-based CT scan segmentation to expedite accurate, repeatable segmentation of whole specimens, improving the scalability of large, comparative studies. While ASP remains a useful metric for the gross characterization of PSP and potential mass savings associated with pneumatization, we anticipate that a focus on bone architectural metrics, coupled with direct mechanical testing of pneumatic and apneumatic bone, will provide more fruitful insights on the functional significance and biomechanical consequences of pneumatization.

The cellular mechanisms and developmental processes governing the onset and extent of pneumatization are essentially unknown, and must be a central focus of future investigation. Several major questions exist on this front. Are the same biochemical actors involved in both cranial and postcranial skeletal pneumaticity? What are the relative contributions of genetic and epigenetic (e.g. biomechanical) influences on the architecture of pneumatic bones? Is biomechanically mediated skeletal pneumatization just a special case of the process of bone functional adaptation that has been documented for primates and other mammals (reviewed in [72]), or does pneumatization have fundamentally distinct impacts on bone architecture? Answering these questions will require experimental design that is sensitive to the myriad, likely interdependent factors that influence skeletal pneumatization.

## Data Availability

New data on regional variation in PSP, as well as all code and input files necessary to repeat the scaling analyses presented here, are available in the electronic supplementary materials. Computed tomography scans used in this study are part of ongoing research on the evolution of lung anatomy and skeletal pneumaticity and will be made publicly available following the completion of those projects. Supplementary material is available online [152].
